# Comparison of Cox Proportional Hazards Regression and Multiple Logistic Regression Models for Identifying Factors Associated With Mortality Among Children With Retinoblastoma in Malaysia: A Retrospective Cohort Study

**DOI:** 10.1002/hsr2.73172

**Published:** 2026-09-07

**Authors:** Zulkifli Nor‐Aizura, Nadiah Wan‐Arfah, Nyi Nyi Naing, Syaratul Emma Hashim, Ismail Shatriah, Ahmad Sukari Ain‐Nasyrah, Norhafizah Hamzah, Jamalia Rahmat, Mohamad Aziz Salowi

**Affiliations:** ^1^ Faculty of Medicine, Universiti Sultan Zainal Abidin Kuala Terengganu Terengganu Malaysia; ^2^ Clinical Research Centre (CRC), Hospital Raja Permaisuri Bainun Ipoh Perak Malaysia; ^3^ Faculty of Health Sciences, Universiti Sultan Zainal Abidin Kuala Terengganu Terengganu Malaysia; ^4^ Department of Ophthalmology and Visual Science School of Medical Sciences, Universiti Sains Malaysia Kubang Kerian Kelantan Malaysia; ^5^ Hospital Pakar Universiti Sains Malaysia Kubang Kerian Kelantan Malaysia; ^6^ Department of Paediatric Ophthalmology Hospital Tunku Azizah, Wilayah Persekutuan Kuala Lumpur Malaysia; ^7^ Department of Ophthalmology Hospital Kuala Lumpur, Wilayah Persekutuan Kuala Lumpur Malaysia; ^8^ Department of Ophthalmology Hospital Selayang, Batu Caves Gombak Selangor Malaysia

**Keywords:** associated factors, Cox proportional hazards regression, mortality, multiple logistic regression, retinoblastoma

## Abstract

**Background and Aims:**

Retinoblastoma is the most common intraocular rare disease in children arising in the retinal cells. This study aimed to evaluate the consistency of the findings of factors associated with mortality in retinoblastoma children between the Cox Proportional Hazards Regression Model (Cox PHR) and the Multiple Logistic Regression Model (MLR).

**Methods:**

A retrospective cohort study was conducted from January 2004 to April 2023 on 402 children diagnosed with retinoblastoma who were admitted to hospitals with paediatric ophthalmology services in Malaysia. The statistical analyses used to model the factors associated with mortality were Cox PHR and the MLR. The five key elements evaluated for parameter estimates encompass direction, estimation, precision, significance, and magnitude of risk. The performance of the models was assessed using the Akaike Information Criterion (AIC) and Bayesian Information Criterion (BIC).

**Results:**

Among 402 children, 22 (5.5%) died, 27 (6.7%) were lost to follow‐up, and 353 (87.8%) remained alive. Prolonged delay in diagnosis and non‐compliance with follow‐up were significant factors associated with mortality in both models. In the Cox PHR model, prolonged delay in diagnosis (AHR: 4.45, 95% CI: 1.86–10.62, *p* = 0.001) and non‐compliance with follow‐up (AHR: 5.21, 95% CI: 2.24–12.10, *p* < 0.001) increased mortality risk. Similar findings were observed in the MLR model, with prolonged delay in diagnosis (AOR: 4.73, 95% CI: 1.84–12.15, *p* = 0.001) and non‐compliance with follow‐up (AOR: 9.51, 95% CI: 3.75–24.11, *p* < 0.001) significantly increasing the odds of mortality. Both regression models showed consistent direction, and significant results.

**Conclusion:**

Both Cox PHR and MLR models consistently identified prolonged delay in diagnosis and non‐compliance with follow‐up as factors associated with mortality among children with retinoblastoma.

## Summary

This study contributes to the growing evidence on methods by assessing Cox PHR and MLR models to identify factors associated with mortality in children with retinoblastoma. The results of the study show that both regression models can produce consistent parameter estimates when using high‐quality and reliable data. This evaluation supports more informed methodological choices in future associated studies involving different types of outcomes.

## 1 Introduction

Retinoblastoma is a rare and aggressive form of eye cancer that primarily affects children globally. It is the most common intraocular malignancy in childhood and typically manifests before the age of five [1]. Retinoblastoma can affect vision and lead to death if not treated promptly. Despite advancements in diagnosis and treatment, the disease remains a significant global health burden, particularly in less‐developed and developing countries. While the mortality rate has significantly decreased in developed countries over the past several decades, the prognosis for children with retinoblastoma in many developing regions, especially in Southeast Asia, remains poor compared to other parts of Asia [2].

Previous studies in developed countries have examined associated factors of mortality from retinoblastoma using a multiple logistic regression model (MLR) [3, 4, 5, 6, 7]. These studies have provided valuable insights into the factors influencing patient outcomes. However, limited research has been conducted on examining factors associated with mortality in children with retinoblastoma using a similar approach in developing countries [8, 9, 10]. Based on our literature search, we are unable to find any studies that have employed MLR model to identify factors associated with mortality among children with retinoblastoma in the Malaysian context.

Alternatively, the Cox Proportional Hazards Regression Model (Cox PHR) has been extensively used in cancer research to analyse survival outcomes and identify factors associated with time‐to‐event data [11]. This semiparametric regression technique evaluates the association between survival time and a set of variables, making it particularly suitable for studying mortality risk in oncology [12]. In the context of survival analysis, an event can encompass various outcomes such as mortality, disease recurrence, the onset of illness, or any other clinically relevant experience that children may undergo. The clinical researcher opted for the Cox PHR model as it can accommodate censored data observations. Censored cases refer to children with incomplete survival time measurements, such as those who did not experience events by the end of the study period, were lost to follow‐up, or experienced a different event of interest [13].

The selection of an appropriate statistical method should be guided by the study design, study objectives, and outcome of interest. In prospective clinical trials with predefined outcomes and standardized follow‐up schedules, conventional regression methods such as logistic or linear regression are commonly used to evaluate treatment effects because follow‐up is relatively uniform and censoring is minimal [14]. In contrast, retrospective cohort studies frequently involve variable follow‐up durations and censored observations, making survival analysis using Cox proportional hazards regression particularly suitable for modeling time‐to‐event outcomes. Nevertheless, MLR remains a widely used approach for analyzing binary mortality outcomes and may provide comparable estimates under certain study conditions.

Cox PHR and MLR models are widely employed in epidemiological and clinical studies to analyse mortality outcomes [15]. In the present study, both regression models were employed to identify factors associated with mortality in children with retinoblastoma using different approaches. While the previous study focused primarily on identifying prognostic factors associated with mortality using Cox PHR [16], the present study represents a distinct secondary analysis that specifically evaluates Cox PHR and MLR models for identifying factors associated with mortality. Thus, this current study further evaluates the consistency of the findings between the two approaches in terms of the direction, estimation, precision, significance, and magnitude of the parameter estimates and determines their model performance.

## 2 Materials and Methods

### 2.1 Study Design and Patient Selection

Data were obtained from children with retinoblastoma who were admitted to paediatric ophthalmology services in various Malaysian hospitals, including Hospital Kuala Lumpur, Hospital Tunku Azizah in Kuala Lumpur, Hospital Wanita dan Kanak‐Kanak Sabah, Hospital Queen Elizabeth in Sabah, Hospital Sultanah Nur Zahirah in Terengganu, Hospital Umum Kuching in Sarawak, and Hospital Pakar Universiti Sains Malaysia in Kelantan. The study population consisted of all children diagnosed with retinoblastoma and admitted to these hospitals between January 2004 and April 2023.

Given the rarity of retinoblastoma, the total population was applied for this study due to the limited number of available cases. The retrospective cohort study included 402 children with retinoblastoma.

### 2.2 Data Source and Eligibility Criteria

Data on children with retinoblastoma were obtained from the Retinoblastoma Registry of the National Eye Database (NED) and hospital medical records using a standardized data collection sheet. This information included the patient's socio‐demographic data, clinical characteristics, treatment modalities, and outcomes. For children with bilateral retinoblastoma, the analysis considered only the eye with the more advanced clinical staging presentation.

All children were clinically diagnosed with retinoblastoma through indirect ophthalmoscopy, and the diagnosis was supported by computed tomography scans or magnetic resonance imaging. For more advanced cases, histopathological examination was used to verify the diagnosis. Children were excluded from the study if they had incomplete information.

The dependent variables in this study were selected based on the chosen statistical analysis. The outcome of interest was mortality, defined as death due to retinoblastoma or related complications. For the Cox PHR model, the dependent variable was time‐to‐event data, with survival time measured in months from the date of diagnosis to the date of death or the date of the last follow‐up visit for censored cases. Children alive at the end of the study period or lost to follow‐up were considered censored observations. In contrast, the dependent variable for the MLR model was a dichotomous outcome of alive or dead children.

### 2.3 Statistical Analysis

Cox PHR and the MLR were used to model factors associated with mortality because both are well‐established multivariable statistical methods for investigating mortality in cancer research. A preliminary analysis was conducted to screen potential factors associated with mortality in children with retinoblastoma. Variables with *p* < 0.250 were used as the predefined screening criteria. Variables meeting this criteria, together with variables considered clinically relevant based on previous evidence, were selected for inclusion in the multivariable regression models. The preliminary main effect model was then obtained upon completion of the variable selection process. All the methods were employed for variable selection, and possible multicollinearity and two‐way interactions between independent variables were thoroughly examined to obtain the preliminary final model. The most parsimonious model was then selected.

Scaled and non‐scaled Schoenfeld tests, and C‐statistics were performed to check for the Cox PHR model assumptions. Regression diagnostics were also conducted, using Cox‐Snell residuals to evaluate model fitness. Any potential outlier was identified through influential diagnostics. An outlier was deemed influential if its removal resulted in a change of 20% or more in the regression coefficient. The final model was expressed based on variables identified, adjusted regression coefficient (β), adjusted hazard ratio (AHR) with 95% confidence interval (CI), and corresponding *p* values.

The overall fit of the MLR model was assessed through the Hosmer‐Lemeshow test, Pearson chi‐square test, correctly classification performance, and the area under the receiver operating characteristic (ROC) curve. Covariate patterns are identified as influential to the model if changes in the regression coefficient of 20% or more were observed. The final model was expressed based on variables identified, adjusted regression coefficient (β), adjusted odds ratio (AOR) with 95% CI, and corresponding *p* values.

The assessment of these two statistical models utilizing different types of dependent variables was based on the five parameter estimates. The first element, which was the direction of the regression coefficient, was related to the risk estimates. A positive regression coefficient indicates a positive relationship with mortality, while a negative regression coefficient indicates a protective effect. The second element was estimation, using a 95% CI to extrapolate from sample statistics to population parameters. Precision, as indicated by the width of the CI, was the third element. The next element is the statistical significance, which was derived through statistical analysis rooted in hypothesis and statistical testing. The *p* value was used to evaluate the statistical significance. Finally, the magnitude of risk was assessed using AHR and AOR to determine how far these risk estimates deviated from the null hypothesis.

The performance of the models was then assessed using the Akaike Information Criterion (AIC) and Bayesian Information Criterion (BIC).

Internal validation of the final MLR model was performed using bootstrap resampling with 1000 replications. In each replication, observations were sampled with replacement from the original dataset and the final multivariable MLR model was refitted. Bootstrap 95% CIs were obtained and compared with the corresponding CIs from the original model to assess the stability and robustness of the regression estimates.

Sensitivity analysis was performed to assess the robustness of the MLR findings to the classification of patients lost to follow‐up. The primary analysis classified the 27 patients lost to follow‐up as alive, coded as 0. Two additional analyses were conducted, first, excluding patients lost to follow‐up from the MLR analysis, second, classifying all patients lost to follow‐up as deaths, coded as 1. The same covariates and modelling strategy were applied across all analyses. All statistical tests were two‐sided with a significance level of 0.050. All analyses were performed using STATA/SE software version 15.

### 2.4 Ethical Consideration

This study was approved by the Medical Research Ethics Committee, Ministry of Health Malaysia (NMRR ID‐22‐00956‐GJE (IIR)), UniSZA Human Research Ethics Committee (UniSZA/UHREC/2023/495(1), and the USM Human Research Ethics Committee (USM/JEPeM/KK/24030236). The requirement for individual informed consent was waived due to the retrospective nature of the data used in this study. However, permission to access patients' medical records was obtained from the participating hospitals. Confidential codes were used on the data collection sheet to represent each patient and protect their privacy.

## 3 Results

A total of 402 children with retinoblastoma were followed up for 20 years and included in the analysis. Of these, 22 children (5.5%) died, while 27 children (6.7%) were lost to follow‐up. The remaining 353 children (87.8%) were alive at the end of the study period. The median age at diagnosis was 23 months (interquartile range: 24 months). Malays ethnicity made up (63.4%) of the total children and a slightly higher proportion of the cohort were males (56.7%). The demographic characteristics of children with retinoblastoma are shown in Table 1.

**Table 1:** Demographic characteristics of children with retinoblastoma.

Variables	Description	Frequency = 402 (%)
Age at diagnosis	Less than 12 months	103 (25.6)
	13–24 months	117 (29.1)
	25–36 months	92 (22.9)
	More than 36 months	90 (22.4)
Ethnicity	Non‐malay	147 (36.6)
	Malay	255 (63.4)
Gender	Female	174 (43.3)
	Male	228 (56.7)
Years of diagnosis	2004–2008	84 (20.9)
	2009–2013	90 (22.4)
	2014–2018	104 (25.9)
	2019–2023	124 (30.8)

Table 2 describes the clinical characteristics of children with retinoblastoma. The most common presenting sign was leukocoria, observed in 74.1% of cases, followed by strabismus (13.0%). Most children (64.9%) presented with unilateral retinoblastoma, while the remaining 35.1% had bilateral disease. The majority of the cohort underwent chemotherapy and enucleation (30.6%). About 13.9% of the children were non‐compliant with follow‐up.

**Table 2:** Clinical characteristics of children with retinoblastoma.

Variables	Description	Frequency = 402 (%)
Family history	Yes	15 (3.7)
	No	387 (96.3)
Leading sign	Leukocoria	298 (74.1)
	Strabismus	52 (13.0)
	Proptosis	21 (5.2)
	Others	31 (7.7)
Delay in diagnosis	Less than 6 months	282 (70.1)
	More than 6 months	120 (29.9)
Laterality	Unilateral	261 (64.9)
	Bilateral	141 (35.1)
Tumor extension	Intraocular	348 (86.6)
	Extraocular	54 (13.4)
Staging	Intraocular A‐B	11 (2.7)
	Intraocular C‐D	84 (20.9)
	Intraocular E	253 (62.9)
	Extraocular	54 (13.5)
Treatment modalities	Single	169 (42.0)
	Multimodalities	233 (58.0)
Treatment received	Enucleation	106 (26.4)
	Chemotherapy	38 (9.5)
	Focal therapy	8 (2.0)
	Chemotherapy + Focal therapy	49 (12.2)
	Chemotherapy + Enucleation	123 (30.6)
	Chemotherapy + EBRT	4 (1.0)
	Chemotherapy + Enucleation + Focal therapy	54 (13.4)
	Chemotherapy + Enucleation + EBRT	10 (2.5)
	Chemotherapy + Enucleation + Focal therapy + EBRT	10 (2.5)
Compliance with follow‐up	Not compliance	56 (13.9)
	Compliance	346 (86.1)
Recurrence	Yes	20 (5.0)
	No	382 (95.0)
Cause of death	Disease progression	14 (63.6)
	Severe sepsis	5 (22.8)
	Unknown	3 (13.6)

Abbreviation: EBRT, external beam radiotherapy.

Table 3 presents the results of the univariable Cox PHR and MLR of factors associated with mortality. A total of eight candidate variables were evaluated during the initial screening process. Three variables were subsequently included in both the final Cox PHR model and MLR model. In the Cox PHR analysis, delay in diagnosis (*p* = 0.001), recurrence (*p* = 0.138), and compliance with follow‐up (*p* < 0.001) were considered candidates for inclusion in the multivariable Cox PHR model. Similarly, in the MLR analysis, delay in diagnosis (*p* = 0.001), recurrence (*p* = 0.069), and compliance with follow‐up (*p* < 0.001) were considered candidates for inclusion in the multivariable MLR model.

**Table 3:** Comparison of associated factors with mortality in retinoblastoma using the univariable regression models.

Variables	Cox proportional hazards regression model			Multiple logistic regression model		
	Regression coefficient (β)	CHR (95% CI)	*p* value	Regression coefficient (β)	COR (95% CI)	*p* value
Age at diagnosis						
Less than 12 months	0	1		0	1	
13–24 months	1.19	3.29 (0.90–11.94)	0.071	1.14	3.12 (0.83–11.65)	0.091
25–36 months	0.47	1.59 (0.36–7.12)	0.542	0.42	1.52 (0.33–6.96)	0.593
More than 36 months	0.73	2.08 (0.50–8.68)	0.318	0.67	1.96 (0.46–8.45)	0.366
Gender						
Female	0	1		0	1	
Male	−0.06	0.94 (0.41–2.18)	0.892	‐0.09	0.91 (0.38–2.16)	0.833
Ethnicity						
Non‐malay	0	1		0	1	
Malay	−0.30	0.74 (0.32–1.73)	0.487	‐0.19	0.82 (0.34–1.98)	0.664
Family history						
No	0	1		0	1	
Yes	0.26	1.30 (0.18–9.69)	0.796	0.22	1.25 (0.16–9.92)	0.836
Laterality						
Bilateral	0	1		0	1	
Unilateral	0.24	1.27 (0.52–3.11)	0.607	0.15	1.17 (0.46–2.93)	0.742
Delay in diagnosis						
Less than 6 months	0	1		0	1	
More than 6 months	1.54	4.69 (1.96–11.18)	0.001	1.51	4.52 (1.84–11.09)	0.001
Recurrence						
No	0	1		0	1	
Yes	0.92	2.51 (0.74–8.51)	0.138	1.22	3.37 (0.91‐12.51)	0.069
Compliance with follow‐up						
Compliance	0	1		0	1	
Not compliance	1.71	5.50 (2.37–12.77)	< 0.001	2.22	9.16 (3.74–22.45)	< 0.001

Abbreviations: CHR, crude hazards ratio; CI, confidence interval; COR, crude odds ratio.

The final model included two estimated parameters and 22 mortality events, corresponding to 11 events per parameter. No evidence of substantial multicollinearity was identified. Clinically plausible interaction terms were also assessed, and no statistically significant interactions were detected in both models.

**Figure 1:**
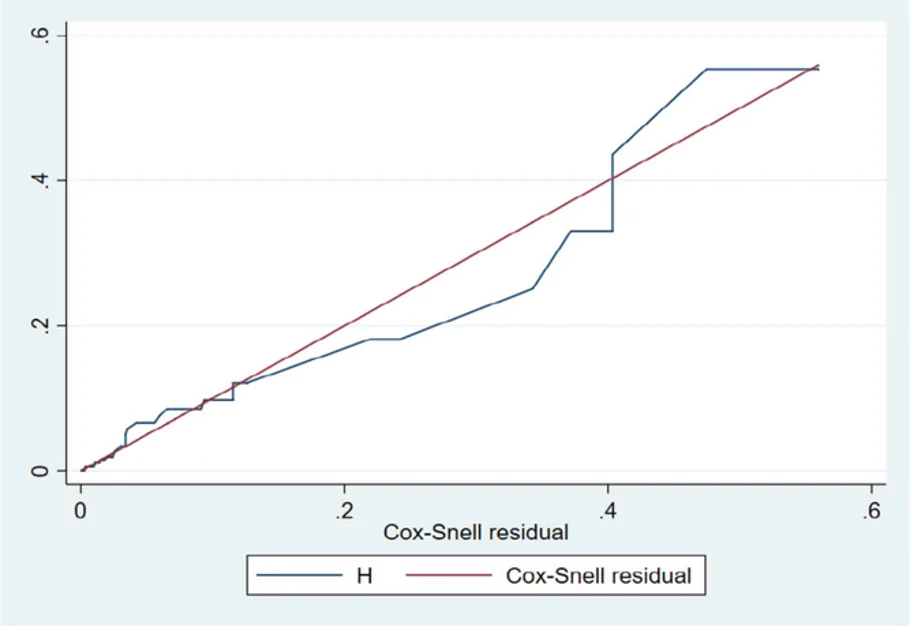
Cox‐Snell residuals of retinoblastoma patients.

The proportional hazards assumption was assessed using scaled Schoenfeld residual tests and global test as indicated in Table 4. No significant evidence of violation of the proportional hazards assumption was observed for delay in diagnosis (*p* = 0.467) and compliance with follow‐up (*p* = 0.140). The global test was also non‐significant (*p* = 0.268), indicating no evidence of time‐dependent departures from the proportional hazards assumption. The Harrell's C‐statistic was 0.758, indicating good discriminatory ability of the Cox proportional hazards model, with a 75.8% concordance between the predicted risk ranking and the observed survival outcomes. The Cox‐Snell residual plot showed that the estimated cumulative hazard generally followed the 45° reference line, suggesting that the Cox proportional hazards model provided a reasonable fit to the observed data as display in Figure 1.

**Table 4:** Scaled and unscaled Schoenfeld residual test.

Variables	Chi‐square	*p* value
Delay in diagnosis	0.53	0.467
Compliance with follow‐up	2.18	0.140
**Global test**	**2.63**	**0.268**

Table 5 presents the model fit and performance measures of the MLR model. The Hosmer‐Lemeshow test indicated no evidence of lack of fit (*p* = 0.894). This was further supported by the Pearson chi‐Square test (*p* = 0.636). The model correctly classified 94.5% of patients overall. The ROC curve demonstrated good discriminatory ability, with an area under the curve of 0.792 (95% CI: 0.69–0.90). Influence diagnostics showed no substantial changes in the regression coefficients attributable to individual observations. Thus, no remedial measures were considered necessary in both models.

**Table 5:** Model fit and performance measures.

Overall fit tests	Results
Hosmer‐Lemeshow	*p* value = 0.894
Pearson‐chi square	*p* value = 0.636
Classification performance	94.5%
Area under the ROC curve	0.792 (95% CI: 0.69, 0.90)

Abbreviation: ROC, receiver‐operating characteristic.

Table 6 shows the statistical regression between the final Cox PHR and the MLR models. The multivariable analysis using the backward selection method retained two significant associated factors in both final models. The analysis identified prolonged delay in diagnosis (*p* = 0.001) and non‐compliance with follow‐up (*p* < 0.001) as significant factors associated with mortality in retinoblastoma children across both models. In the Cox PHR model, children with a delay in diagnosis of more than 6 months had a significantly 4.45 times higher risk of mortality than children with less than 6 months (AHR: 4.45, 95% CI: 1.86–10.62, *p* = 0.001). Similarly, the MLR model indicated that a delay in diagnosis of more than 6 months was associated with 4.73 times the odds of mortality (AOR: 4.73, 95% CI: 1.84–12.15, *p* = 0.001). Moreover, in the Cox PHR model, children non‐compliant with follow‐up had a 5.21 times higher risk of mortality compared to those who were compliant (AHR: 5.21, 95% CI: 2.24–12.10, *p* < 0.001). Similarly, the MLR analysis indicated that non‐compliance with follow‐up was associated with 9.51 times the odds of mortality (AOR: 9.51, 95% CI: 3.75–24.11, *p* < 0.001).

**Table 6:** Comparison of factors associated with mortality in retinoblastoma using the multivariable regression models.

Variables	Cox proportional hazards regression model			Multiple logistic regression model		
	Regression coefficient (β)	AHR (95% CI)	*p* value	Regression coefficient (β)	AOR (95% CI)	*p* value
Prolonged delay in diagnosis	1.49	4.45 (1.86–10.62)	0.001	1.55	4.73 (1.84–12.15)	0.001
Non‐compliance with follow‐up	1.65	5.21 (2.24–12.10)	< 0.001	2.25	9.51 (3.75–24.11)	< 0.001

Abbreviations: AHR, adjusted hazards ratio; AOR, adjusted odds ratio; CI, confidence interval.

The two models demonstrated consistent findings for prolonged delay in diagnosis and non‐compliance with follow‐up. Both variables showed a positive direction of association, with positive regression coefficients and estimates greater than one, indicating increased mortality hazard in the Cox PHR model and increased mortality odds in the MLR model. The variables were significant in both models. For prolonged delay in diagnosis, the 95% CIs were 1.86–10.62 in the Cox PHR model and 1.84–12.15 in the MLR model, while for non‐compliance with follow‐up, the corresponding 95% CIs were 2.24–12.10 in the Cox PHR model and 3.75–24.11 in the MLR model, respectively. Prolonged delay in diagnosis was associated with a 4.45‐fold higher hazard of mortality in the Cox PHR model and 4.73‐fold higher odds of mortality in the MLR model, while for non‐compliance with follow‐up was associated with a 5.21‐fold higher hazard of mortality in the Cox PHR model and 9.51‐fold higher odds of mortality in the MLR model, respectively. The comparison of parameter estimates using the multivariable regression models is shown in Table 7.

**Table 7:** Comparison of parameter estimates using multivariable regression models.

Variables	Elements	Cox proportional hazards regression	Multiple logistic regression
Prolonged delay in diagnosis	Direction	*β* = 1.49; AHR > 1	*β* = 1.55; AOR > 1	
	Estimation	*e* ^1.49^ = 4.45	*e* ^1.55^ = 4.73	
	Precision	95% CI = 1.86–10.62	95% CI = 1.84–12.15	
	Significance	*p* value = 0.001	*p* value = 0.001	
	Magnitude	4.45‐fold higher hazard	4.73‐fold higher odds	
Non‐compliance with follow up	Direction	*β* = 1.65; AHR > 1	*β* = 2.25; AOR > 1
	Estimation	*e* ^1.65^ = 5.21	*e* ^2.25^ = 9.51	
	Precision	95% CI = 2.24–12.10	95% CI = 3.75–24.11	
	Significance	*p* value < 0.001	*p* value < 0.001	
	Magnitude	5.21‐fold higher hazard	9.51‐fold higher odds	

Abbreviations: AHR, adjusted hazards ratio; AOR, adjusted odds ratio; β, Regression coefficient.

The AIC and BIC values for the MLR model were 143.81 and 155.80, respectively, while those for the Cox PHR model were 224.07 and 232.06, respectively.

Table 8 shows the internal validation of the MLR model using 1000 bootstrap resamples. The bootstrap CIs were broadly comparable with the corresponding CIs from the original model, supporting the stability of the identified associations.

**Table 8:** Internal validation of the multiple logistic regression model using bootstrap resampling.

Variables	Original analysis AOR (95% CI)	Bootstrap resampling 95% CI	Bootstrap *p* value
Prolonged delay in diagnosis	4.73 (1.84–12.15)	1.62–13.79	0.004
Non‐compliance with follow‐up	9.51 (3.75–24.11)	3.52–25.69	< 0.001

*Note:* Bootstrap internal validation was performed using 1000 resamples with replacement.

Abbreviations: AOR, adjusted odds ratio; CI, confidence interval.

Sensitivity analysis was performed to assess the robustness of the MLR findings to alternative classifications of the 27 patients lost to follow‐up. In the primary analysis, patients lost to follow‐up were classified as non‐deaths. When these patients were excluded from the analysis, the associations between prolonged delay in diagnosis and mortality (AOR: 6.35, 95% CI: 2.10–19.42, *p* = 0.001) and non‐compliance with follow‐up and mortality (AOR: 7.75, 95% CI: 2.64–22.72, *p* < 0.001) remained statistically significant. Similarly, when all patients lost to follow‐up were classified as deaths, prolonged delay in diagnosis (AOR: 4.36, 95% CI: 2.23–8.51, *p* < 0.001) and non‐compliance with follow‐up (AOR: 3.71, 95% CI: 1.75–7.83, *p* = 0.001) remained significantly associated with mortality as shown in Table 9.

**Table 9:** Sensitivity analysis of the multiple logistic regression model under alternative classifications of patients lost to follow‐up.

Variables	Primary analysis: LTFU classified as non‐death (*n* = 402)		Sensitivity analysis 1: LTFU excluded (*n* = 375)		Sensitivity analysis 2: LTFU classified as death (*n* = 402)	
	AOR (95% CI)	*p* value	AOR (95% CI)	*p* value	AOR (95% CI)	*p* value
Prolonged delay in diagnosis	4.73 (1.84–12.15)	0.001	6.35 (2.10–19.42)	0.001	4.36 (2.23–8.51)	< 0.001
Non‐compliance with follow‐up	9.51 (3.75–24.11)	< 0.001	7.75 (2.64–22.72)	< 0.001	3.71 (1.75–7.83)	0.001

Abbreviations: AOR, adjusted odds ratio; CI, confidence interval; LTFU, lost to follow‐up.

## 4 Discussion

Our comprehensive literature review did not identify any studies that have assessed the performance of the Cox PHR and the MLR models in identifying factors associated with mortality among retinoblastoma children. Previous studies have evaluated the parameter estimates generated by similar statistical models [17, 18, 19, 20, 21, 22]. Additionally, several studies have explored and compared the performance of various statistical models in estimating parameters [23, 24, 25]. The reported 5% mortality rate among children with retinoblastoma provides a crucial benchmark for evaluating outcomes in this rare but life‐threatening disease [4]. Timely diagnosis and appropriate treatment, with a focus on eliminating extraocular tumour, are essential for improving survival outcomes for the children. Recent evidence suggests that intra‐arterial chemotherapy may offer favorable globe‐salvage and enucleation‐avoidance outcomes compared with intravenous chemotherapy, highlighting the importance of treatment modality and disease stage in retinoblastoma outcomes [26].

Clinical research examining associations frequently employed Cox PHR and MLR models for data analysis [27]. A Cox PHR model accounts for the duration of follow‐up and assesses whether a risk factor affects the timing of the disease event. The fundamental assumption of this model is that the AHR between the two groups remains constant over time [28]. However, failing to assess the proportional hazards assumption can lead to misinterpretation of the findings. Additionally, the MLR model evaluates whether a risk factor influences the odds of the disease, without considering the timing of the event. As a result, early and late events are weighted equally in the MLR model. The assumption is that all individuals will eventually experience the event, but it is not observed for some within the follow‐up period [17]. The AOR is used to predict a binary outcome based on a set of independent factors, which can be either continuous or categorical data [29]. Thus, it is reasonable that the results would differ, as observed in the current study.

Both Cox PHR and MLR models consistently identified prolonged delay in diagnosis and non‐compliance with follow‐up as significant factors associated with mortality. The consistency in terms of direction, with positive direction greater than one, indicating increased in risk, and significance demonstrates that these variables are robust, regardless the statistical method employed. The observed differences between AHRs and AORs are expected because the Cox PHR model estimated the instantaneous risk of mortality over time by accounting for censoring. In contrast, the MLR model estimates the odds of mortality without considering the follow‐up period.

The sensitivity analysis demonstrated that the associations between prolonged delay in diagnosis and non‐compliance with follow‐up and mortality remained statistically significant when patients lost to follow‐up were excluded or classified as deaths. Although the magnitude of the associations varied across the different classifications, the direction and statistical significance of the associations remained consistent, supporting the robustness of the primary findings. Although advances in predictive modelling have demonstrated promising performance in ophthalmic disease, recent evidence emphasises the need for cautious interpretation and further validation before clinical implementation, particularly when evidence is derived from a limited number of studies or populations [30].

This finding is consistent with the comparable parameter estimates obtained from both models, indicating that the MLR model identified the same significant factors associated with mortality while providing an adequate fit to the observed data. Although the Cox PHR model remains the preferred approach for analysing time‐to‐event data, as it accounts for follow‐up times and censoring, the MLR model in this study demonstrated acceptable discrimination, and bootstrap analysis supported the stability of its estimates. The clinical applicability of statistical models should also be considered in relation to the setting in which they are developed and evaluated. Recent evidence has demonstrated that diagnostic performance may vary across clinical pathways and according to disease prevalence, decision thresholds, and the consequences of misclassification [31]. Moreover, the larger longitudinal studies are needed to validate the findings and translate them into routine practice [32].

The current findings are consistent with a previous study on caesarean delivery and its associated factors, which reported that some factors were consistently identified across both Cox PHR and MLR models [18]. The difference in model performance may reflect characteristics of the study population, including outcome prevalence, event frequency, and the strength of associations between determinants and outcomes, which can influence the performance and estimates of different statistical models. In addition, a recent retrospective study on factors associated with steroid‐induced ocular hypertension in children with systemic lupus erythematosus used the MLR model to analyze a binary outcome, providing a parallel example of the usability of logistic regression in such studies of paediatric ophthalmic factors [33].

In contrast, studies examining genetic associations with disease outcomes demonstrated that Cox PHR model produced more reliable and precise estimates than MLR model [19]. Similarly, analysis of the 26‐year Framingham Heart Study showed that Cox PHR model better utilized longitudinal time‐to‐event information [20]. In addition, another study from France reported better performance of Cox PHR model in identifying risk factors for neurosurgical site infection following neurosurgery [21].

Regarding future research directions, two aspects can be expanded. First, greater attention could be given to more objective outcome measures. A study using optical coherence tomography to measure ciliary muscle thickness in children suggested a method for quantitative assessment of ocular structural changes [34]. Future research could adopt this technique to extend the prognostic evaluation of children with retinoblastoma from a single mortality endpoint to structural and functional indicators, thereby enriching the outcome evaluation system. Second, the application of artificial intelligence (AI) in fundus image analysis is also promising. A high‐impact study reporting an AI framework for multidisease detection from retinal images demonstrated the feasibility of using deep learning models to screen for systemic diseases [35]. In the future, similar techniques could be attempted to combine fundus imaging with AI to predict prognosis or perform risk stratification in children with retinoblastoma.

In the present study, no evidence of multicollinearity and interaction effects was identified in both models. Furthermore, comprehensive diagnostic assessments indicated that both models satisfied the respective assumptions. For the Cox PHR model, there was no evidence of violation of the proportional hazards assumption was observed, and the regression diagnostics demonstrated an adequate model fit without influential outliers. Similarly, the MLR model exhibited satisfactory goodness of fit and acceptable diagnostic performance, with no evidence of influential outliers that substantially affected the regression estimates. Thus, these results increase confidence that the identified associated factors were not influenced by model misspecification or unstable parameter estimates.

The selection between the Cox PHR and MLR models should be guided by the study design, study objectives, the nature of the outcome variable, and the underlying model assumptions. The Cox PHR model is generally preferred for time‐to‐event data because it accounts for varying follow‐up durations and censored observations. In contrast, the MLR model is an appropriate approach when the outcome is binary and survival time is not the primary focus. In addition, future research could consider parametric survival models or machine learning methods and conduct broader comparisons with the Cox PHR model.

The study may be subject to loss to follow‐up bias. Loss to follow‐up bias could arise when patients discontinue monitoring before the study endpoint, potentially limiting the accuracy of survival outcomes. Furthermore, the exclusion of observations with a potentially worse prognosis could have led to an underestimation of potential confounding factors, potentially influencing the identifying factors associated with mortality in the models. The large sample size, which accounted for the majority of retinoblastoma cases observed across hospitals with specialised paediatric ophthalmology services in Malaysia, enhances the generalizability and significance of the study findings.

## 5 Conclusion

Both Cox PHR and MLR models consistently identified prolonged delay in diagnosis and non‐compliance with follow‐up as factors associated with mortality among children with retinoblastoma. The consistency of these findings across different approaches supports their potential prognostic relevance. These findings suggest that different statistical regression models can yield comparable and robust results when high‐quality and reliable sources are utilised.

## Author Contributions

**Zulkifli Nor‐Aizura:** conceptualization, methodology, resources, data curation, formal analysis, original draft. **Nadiah Wan‐Arfah:** conceptualization, methodology, formal analysis, supervision, review, validation. **Nyi Nyi Naing:** conceptualization, methodology, formal analysis, supervision, review, validation. **Syaratul Emma Hashim:** conceptualization, methodology, supervision, review, validation. **Ismail Shatriah:** resources, data curation, supervision, review, validation. **Ahmad Sukari Ain‐Nasyrah:** resources, data curation, review, validation. **Norhafizah Hamzah:** resources, data curation, review, validation. **Jamalia Rahmat:** methodology, resources, data curation, review. **Mohamad Aziz Salowi:** resources, data curation, review.

## Disclosure

All authors have read and approved the final version of the manuscript Nadiah Wan‐Arfah, had full access to all of the data in this study and takes complete responsibility for the integrity of the data and the accuracy of the data analysis.

## Ethics Statement

The authors stated that the study received approval from the Medical Research & Ethics Committee of Universiti Sultan Zainal Abidin ((UniSZA/UHREC/2023/495(1)), Universiti Sains Malaysia (USM/JEPeM/KK/24030236), and the Ministry of Health Malaysia (NMRR ID‐22‐00956‐GJE (IIR)).

## Consent

Written informed consent was not required as the study was conducted retrospectively.

## Conflicts of Interest

The authors declare no conflicts of interest.

## Transparency Statement

The lead author, Nadiah Wan‐Arfah, affirms that this manuscript is an honest, accurate, and transparent account of the study being reported; that no important aspects of the study have been omitted; and that any discrepancies from the study as planned (and, if relevant, registered) have been explained.

## Data Availability

The data that support the findings of this study are available on request from the corresponding author. The data are not publicly available due to privacy or ethical restrictions.
